# The way we encounter reading material influences how frequently we mind wander

**DOI:** 10.3389/fpsyg.2013.00892

**Published:** 2013-11-28

**Authors:** Trish L. Varao Sousa, Jonathan S. A. Carriere, Daniel Smilek

**Affiliations:** Department of Psychology, University of WaterlooWaterloo, ON, Canada

**Keywords:** reading silently, reading aloud, listening, mind wandering, memory, interest ratings

## Abstract

We examined whether different encounters of reading material influence the likelihood of mind wandering, memory for the material, and the ratings of interest in the material. In a within-subjects design participants experienced three different reading encounters: (1) reading a passage aloud, (2) listening to a passage being read to them, and (3) reading a passage silently. Throughout each reading encounter probes were given in order to identify mind wandering. After finishing the passage participants also rated how interesting it was and completed a content recognition test. Results showed that reading aloud led to the least amount of mind wandering, while listening to the passage led to the most mind wandering. Listening to the passage was also associated with the poorest memory performance and the least interest in the material. Finally, within the silent reading and listening encounters we observed negative relations between mind wandering and both memory performance and interest in the material, replicating previous findings. Taken together, the present findings improve our understanding of the nature of mind wandering while reading, and have potentially important implications for readers seeking to take advantage of the convenience of audiobooks and podcasts.

## Introduction

We live in an age defined by rapid technological change, and one of the areas in which new technology has affected us most is the electronic distribution of information. While most of us first learned to understand the world outside our homes by reading newspapers, magazines, and books, electronic versions are already rapidly replacing these traditional media—and even those are giving way to newer, more convenient, methods of information consumption, such as podcasts (digital media) and audiobooks (Pierleoni, 2011; Houston Santhanam et al., 2012; Business Wire, 2013). Each of these new methods changes the ways in which individuals interact with and, potentially, process information. Interestingly, the trend seems to be toward putting less active effort into our information consumption, and limiting the degree to which we must physically engage with the material. Indeed, listening to an audiobook, for example, requires aural effort alone and, compared the previous default of silently reading our books or newspapers, frees up our eyes so they can be put to use elsewhere. A seemingly natural consequence of this freedom, however, is that one's ability to stay focused on the task may be jeopardized, as the mind begins to wander off to other information that happens to capture the attention of our unencumbered eyes. This suggests that different ways of engaging with information might coopt attention to different degrees, and thus might affect both how much time one spends mind wandering and, ultimately, how well the information is remembered. To examine these we directly compare the influences on mind wandering of three natural ways of engaging with reading material, namely: (1) reading silently, (2) reading aloud, and (3) listening.

While our study was mainly exploratory in nature, we did have some general reasons for comparing mind wandering while silently reading, reading aloud, and listening. Importantly, we included in our comparison an examination of mind wandering during *silent reading* primarily because it is clearly still the most common way people engage textual material in everyday life. In addition, many recent studies of mind wandering while reading have taken place in silent reading conditions (e.g., Schooler et al., 2004; Smilek et al., 2010; Franklin et al., 2011; Uzzaman and Joordens, 2011). Indeed, its frequent inclusion in previous studies makes silent reading essentially a necessary baseline condition. As well, silent reading may serve as a particularly effective baseline, given that some studies have found individuals report spending nearly half of their time mind wandering (e.g., Uzzaman and Joordens, 2011). Thus, it should be quite possible for one to spend both more and less time mind wandering, relative to silent reading, and it seems reasonable to assume there would be other ways of encountering reading material that either substantially increase or decrease one's rate of mind wandering.

Taking silent reading as a baseline condition, one of our main goals was to compare rates of mind wandering during silent reading with rates of mind wandering during *reading aloud*. While reading aloud is not very common today, many authors believe that at least until Roman times reading aloud was the norm (Manguel, 1996; Gavrilov, 1997; Saenger, 1997), and it seems monastic scribes were not specifically required to read silently until the 9th century (Manguel, 1996)—suggesting this was a change from the traditional reading behavior at the time. A famous example of this is St. Augustine's surprise at discovering Bishop Ambrose's ability to read silently, in the 4th century AD (Augustine, trans, 1961). When considering reading aloud in comparison to silent reading, it seems reasonable that reading aloud might yield less mind wandering than silent reading. After all, a growing volume of literature has shown that the body is intimately involved in cognition (e.g., Findlay and Gilchrist, 2003; Barsalou, 2008; Hesslow, 2012) and, in combination with the perceptual decoupling hypothesis of mind wandering recently raised by Smallwood and colleagues (e.g., Smallwood, 2013; Smallwood et al., 2013), this view suggests that the more bodily systems one has recruited at any given time the less likely one may be to fully decouple and engage instead in mind wandering. In the context of reading aloud both ocular and aural information translation processes are necessarily engaged, as are the motor processes necessary for speech production. In this regard, reading aloud certainly provides a more fully physically engaged reading experience than silent reading and, thus readers may be more likely to have greater attentional engagement as well. Recent work on the production effect by MacLeod and colleagues (MacLeod et al., 2010; Ozubko et al., 2012) supports this view, as they have shown that reading aloud, compared to reading silently, leads to better memory for the material. Perhaps our ancient ancestors had the right idea, then—reading aloud might be a much better way to engage with and remain attentive to important information, and is worth revisiting as a potential strategy for reducing mind wandering.

Another of our main goals was to directly compare rates of mind wandering across silent reading and *listening* conditions. Given the growing trend toward obtaining information by auditory means alone, such as podcasts and audiobooks, it is important to consider whether their obvious convenience comes at a cost to attention and mind wandering. Notably, unlike silent reading (and reading aloud), if one is simply listening to a podcast or pre-recorded lecture, only aural engagement is required, and no other environmental feedback is available. This could be problematic for the listener, as oculomotor and other feedback processes may both facilitate maintaining attention on the task at hand and make moments of inattention more salient. During simple listening tasks, then, one may easily begin to shift to merely hearing^1^ during mind wandering, without noticing it, and ultimately experience more prolonged mind wandering episodes. Consistent with this speculation, studies of listening to lectures suggest that superficial listening or mind wandering are quite common in such situations (e.g., 40% of the time in Cameron and Giuntoli, 1972; 32.9% in Lindquist and McLean, 2011; 39 and 43% in Risko et al., 2012; 41% in Szpunar et al., 2013). In contrast, studies of silent reading have often found substantially less mind wandering (e.g., 9% in Reichle et al., 2010; 19% in Smallwood et al., 2009; 23% in Schooler et al., 2004), though similar and even higher rates have also been reported (e.g., 48% in Krawietz et al., 2012; 32.5 and 37.5% in Smilek et al., 2010; 49% in (Uzzaman and Joordens, 2011)). These findings may suggest that mind wandering occurs at a higher rate during listening than during silent reading, however, the research is clearly far from conclusive. Importantly, very different settings and materials have been used across these studies, leaving open the question of whether, when controlling for these factors, rates of mind wandering are indeed greater during listening than silent reading.

In addition to assessing the rate of mind wandering across conditions, we were interested in studying other potentially relevant outcomes that have been considered in previous research. The most obvious cost likely to be associated with mind wandering is reduced material retention. In previous studies, memory performance has been measured via multiple choice tests following silent reading or viewing and listening to lecture material (Schooler et al., 2004; Smallwood et al., 2008; Franklin et al., 2011; Risko et al., 2012), as well as via end-of-term grades for some in-class experiments (Lindquist and McLean, 2011). These studies have all revealed significant negative correlations, demonstrating that as mind wandering reports increase test performance tends to decrease. We provided participants with a multiple choice memory test for the material following their encounter with each passage in hope of replicating these findings. An additional outcome we were interested in was participants' ratings of their interest in the material, and so we also asked participants to rate their interest in each passage. Giambra and Grodsky (1989) previously investigated the possibility that one's interest in reading material may influence the frequency of mind wandering, and they found that greater interest in the material was associated with less mind wandering. Notably, Giambra and Grodsky manipulated interest by using different reading materials, and thus the material itself could have been the cause of increased mind wandering, rather than participants' interest in it. Nonetheless, such findings suggest that if the way in which our participants encounter the material influences their mind wandering, as we expect, then we may also find similar influences on their interest in the material.

In summary, we examined mind wandering, memory performance and interest ratings across three different reading conditions: silent reading, reading aloud, and listening. We used a within-subjects design in which all participants were exposed to 3 separate passages: one read silently, one read aloud, and one listened to. During each encounter participants were presented with mind wandering probes, used to assess differences in frequency of mind wandering. After each encounter of a passage, participants were asked to rate their interest in the passage on a five-point scale, and they were then given a multiple choice comprehension test examining their memory for the material. We also evaluated the robustness of our admittedly exploratory findings by collecting data from two separate samples of participants, and comparing our findings across the samples.

## Methods

### Participants

As our study was exploratory in nature, two large independent samples of participants were collected in order to replicate and confirm each of our findings. The two samples were collected across two terms in one academic year and no changes were made between the samples. A total of 235 University of Waterloo students participated in return for course credit (Sample 1: 114; Sample 2: 121). A total of 24 participants (Sample 1: 8 participants; Sample 2: 16) were dropped from further analyses for failure to complete the task as required (i.e., did not read aloud when instructed to, or skipped through reading material and therefore did not receive any probes). No additional participant or data exclusions were made. Student ages ranged from 17 to 31 years (Sample 1: *M*_age_ = 19.8 years, *SD* = 2.15, Sample 2: *M*_age_ = 19.9 years, *SD* = 1.63). Seventy five percent of participants were female. Participants were pre-screened to select only those with normal or corrected-to-normal hearing and vision.

### Materials and measures

Stimuli were displayed using an Intel® Atom™ 230 desktop computer, an Intel® Core™ 2 T7200 laptop computer, or an Intel Pentium™ 4 desktop computer with 19 in, 15.3 in, and 19 in displays, respectively. The program was designed using Python 2.6 and Pygame 1.9. When completing the self-reading conditions, whether aloud or silent, participants were presented the passage in black, size 18 Times New Roman font, single-spaced, against a white background slide measuring 1266 × 718 pixels. Participants were presented one page at a time and moved forward to the next page by pressing “n” on the keyboard. When listening to another person read, text stimuli were not displayed and participants were simply asked to keep their eyes focused on a blank white screen while listening. When reading aloud, participants were recorded with a microphone in order to later verify participants had complied with the instructions. The quality of these recordings was sufficient for verifying that participants had read aloud, but did not afford detailed analysis of the recording. No additional manipulations were included in the study.

#### Reading materials

Three excerpts from Bill Bryson's *A Short History of Nearly Everything* (2003) were used. Excerpts from this book have been used in previous studies of mind wandering while reading (Smallwood et al., 2009; Smilek et al., 2010). Each excerpt covered a separate topic and was edited to roughly 1800 words each. In the listening encounter, participants listened to an audio version of the passages read by Bill Bryson.

#### Mind wandering probes

During each reading encounter participants were presented with 10 mind-wandering probes. These were displayed via a blue “pop-up” box in the center of the screen which stated “During the moments prior to the probe, were you mind-wandering?” with key-press response options “1 = Yes 0 = No.” These probes were presented on a 30–90 s randomized schedule. Following participant response the blue “pop-up” box displayed the following message “Task will restart in 3 s… ” after which the pop-up box disappeared and participants could resume the task. Although each experimental task was designed to display 10 probes a number of participants in the silent reading condition completed the task sooner than anticipated, due to faster than average reading times. To account for the absence of some probes across participants and conditions, mind-wandering reports are reported as the proportion of instances of mind wandering for the number of probes received^2^.

#### Memory test

Previous research has shown a negative correlation between mind-wandering reports in silent reading and performance on memory tests related to the material (Schooler et al., 2004; Smallwood et al., 2008; Franklin et al., 2011; Risko et al., 2012). Accordingly, we also asked participants to complete a 10-item True-False memory test following each excerpt (see Appendix). These questions were presented on-screen in black, size 18 Times New Roman font, against a white background slide, and required participants to make responses via a True or False key-press (i.e., T = True, F = False).

#### Interest rating

To both replicate Giambra and Grodsky's (1989) finding that interest was negatively correlated with mind-wandering reports in silent reading tasks, following each excerpt we asked participants to rate their interest in the material on a 5-point scale, where a rating of 1 indicates very little interest (I was NOT interested in this material at all and I did not enjoy reading it) and 5 indicates strong interest (This is the most interesting material I've read in the past year and I would like to read even more on this topic). This scale is the same as was used by Giambra and Grodsky, with the exception of point 2 which was modified as follows: I was only slightly interested in this material and would not like to read further on this topic.

### Procedure

Participants were brought into the lab and provided information related to the study before providing informed consent. The nature of the task was explained with a definition of mind-wandering provided as: “Any thoughts that are experienced that are not related to the material being presented” [based on Lindquist and McLean (2011), p. 161]. Participants were also provided with examples of what mind-wandering could include (e.g., thoughts about dinner; thoughts about past weekend events; concerns about an upcoming exam; thoughts about friends or significant others). Participants were then left to complete the task alone. After each excerpt participants were asked to rate their interest in the material, and then completed a memory test on its content. No additional measures were collected. Upon completion participants were thanked and provided feedback on the experiment. To ensure no order effects of the stimuli presentation would affect results, both encounter types and excerpt order were counterbalanced such that there were 36 possible presentations; however, because a few non-compliant participants were dropped from the analyses, the final samples were only approximately counterbalanced.

## Results

### Mind wandering reports

**Figure 1:**
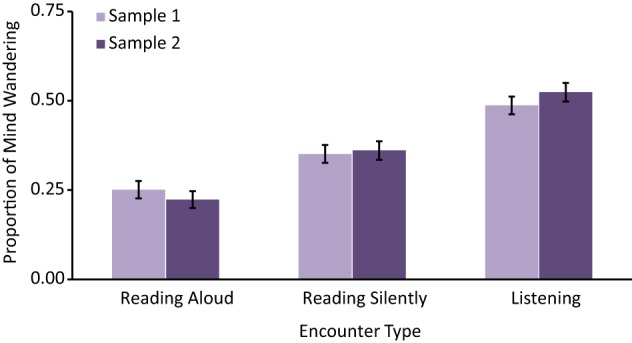
**Mean proportion of mind wandering across reading encounters**. Error bars represent one standard error of the mean.

All analyses were conducted separately for each sample in order to demonstrate the replicability (or lack thereof) of our findings. The average Proportions of Mind Wandering in each Encounter Type, for Samples 1 and 2, are displayed in Figure 1. Here we see similar rates of mind wandering for Listening (*M* = 0.506) and Reading Silently (*M* = 0.356) as reported by other studies (see the Introduction). Repeated measures ANOVAs were first completed to compare the effects of different Encounter Types on Proportions of Mind Wandering in each sample. These analyses confirmed significant main effects of Encounter Type for both Sample 1, *F*_(2, 210)_ = 36.44, MSE = 0.04, *p* < 0.001, η^2^_*p*_ = 0.26, and Sample 2, *F*_(2, 208)_ = 60.91, MSE = 0.04, *p* < 0.001, η^2^_*p*_ = 0.37. Planned follow-up two-tailed paired-samples *t*-test analyses revealed a number of significant differences across Encounter Types. First, significant differences in Proportion of Mind Wandering were found between Reading Aloud and Reading Silently for Sample 1 *t*_(105)_ = 3.50, *p* = 0.001, 95% CI [−0.16 to −0.04], *r* = 0.33, as well as Sample 2, *t*_(104)_ = 5.27, *p* < 0.001, 95% CI [−0.19 to −0.09], *r* = 0.46, such that Reading Silently led to greater mind wandering. Second, Listening led to more mind wandering than Reading Aloud, for Sample 1 *t*_(105)_ = 8.66, *p* < 0.001, 95% CI [0.18–0.29], *r* = 0.65, as well as Sample 2, *t*_(104)_ = 11.17, *p* < 0.001, 95% CI [0.25–0.35], *r* = 0.74. Finally, we also found Listening involved more mind wandering than Reading Silently for both Sample 1 *t*_(105)_ = 4.97, *p* < 0.001, 95% CI [0.08–0.19], *r* = 0.44, and Sample 2, *t*_(104)_ = 5.67, *p* < 0.001, 95% CI [0.11–0.22], *r* = 0.49. The striking similarity of these results suggests there was good replication of findings between the two samples. To test this hypothesis, an omnibus ANOVA was conducted on the rates of mind wandering, including Sample (Samples 1 and 2) as a between participant factor and Encounter Type (Reading Aloud, Reading Silently, and Listening) as a within participant factor. Importantly, the ANOVA yielded a significant effect of Encounter Types, *F*_(2, 418)_ = 95.53, MSE = 0.04, *p* < 0.001, η^2^_*p*_ = 0.31, while the main effect of Sample was not significant (*p* = 0.82) and the interaction between Encounter Type and Sample also was not significant (*p* = 0.25).

### Memory test

**Figure 2:**
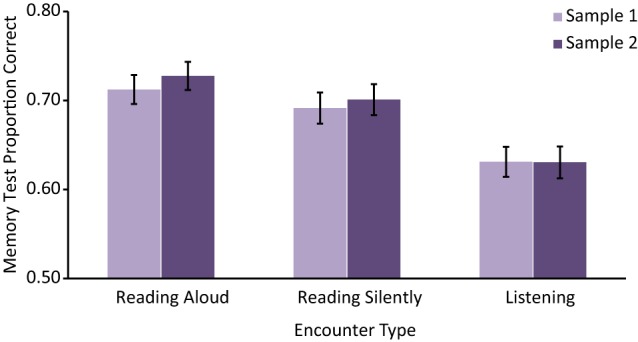
**Mean Memory Test Proportion Correct across reading encounters**. Error bars represent one standard error of the mean.

The average Memory Test Proportion Correct for each Encounter Type and both Samples are displayed in Figure 2. As with mind wandering reports, we performed a set of repeated measures ANOVAs to detect differences in memory scores between reading encounters in each sample, separately. These analyses confirmed significant differences in Memory Test Proportion Correct for both Sample 1, *F*_(2, 210)_ = 7.09, MSE = 0.03, *p* = 0.001, η^2^_*p*_ = 0.06, and Sample 2, *F*_(2, 208)_ = 12.25, MSE = 0.02, *p* < 0.001, η^2^_*p*_ = 0.11. Planned *t*-test analyses revealed significant differences such that Listening led to worse performance than Reading Aloud for both Sample 1, *t*_(105)_ = 3.64, *p* < 0.001, 95% CI [−0.13 to −0.04], *r* = 0.34, and Sample 2, *t*_(104)_ = 4.82, *p* < 0.001, 95% CI [−0.14 to −0.06], *r* = 0.43, and also compared to Reading Silently for Sample 1 *t*_(105)_ = 2.62, *p* = 0.01, 95% CI [−0.11 to −0.01], *r* = 0.25, and Sample 2, *t*_(104)_ = 3.43, *p* < 0.001, 95% CI [−0.11 to −0.03], *r* = 0.32. On the other hand, despite significant differences in Proportion of Mind Wandering, no significant differences were found between memory test scores for Reading Aloud and Reading Silently for either Sample 1, *t*_(105)_ = 0.95, *p* = 0.343, 95% CI [−0.02–0.06] or Sample 2, *t*_(104)_ = 1.33, *p* = 0.188, 95% CI [−0.01–0.07]. Again the results appear to show good replication across the two samples, and an omnibus ANOVA, including Sample as a between participants factor and Encounter Type as a within participant factor, confirmed this view with a significant main effect of Encounter Type, *F*_(2, 418)_ = 18.67, MSE = 0.02, *p* < 0.001, η^2^_*p*_ = 0.08, but neither a significant main effect of Sample (*p* = 0.63) nor a significant interaction of Encounter Type and Sample (*p* = 0.87).

### Interest rating

**Figure 3:**
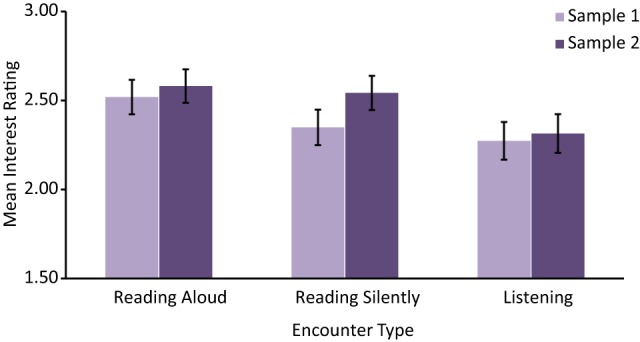
**Mean interest rating across reading encounters**. Error bars represent one standard error of the mean.

The average Interest Ratings in each Encounter Type, for Samples 1 and 2, are displayed in Figure 3. To detect differences in interest in the material across Encounter Type we again conducted a set of repeated measures ANOVAs, one for each sample. Although consistent with one another, both of these analyses only trended toward significance (Sample 1, *F*_(2, 210)_ = 2.35, MSE = 0.711, *p* = 0.098, η^2^_*p*_ = 0.02, and Sample 2, *F*_(2, 208)_ = 2.88, MSE = 0.758, *p* = 0.058, η^2^_*p*_ = 0.03), indicating that the effect of Encounter Type on Interest Rating is minimal, and likely requires a large sample size in order to be detected. Planned follow-up *t*-test analyses revealed only one consistent significant difference across samples, such that Listening led to less interest in the material than Reading Aloud for both Sample 1, *t*_(105)_ = 2.33, *p* = 0.022, 95% CI [−0.45 to −0.04], *r* = 0.22, and Sample 2, *t*_(104)_ = 2.13, *p* = 0.035, 95% CI [−0.51 to −0.02], *r* = 0.21. No other significant differences were found. To determine whether a larger sample could detect an effect of encounter on interest, an omnibus ANOVA was conducted on the Interest Ratings, including Sample as a between participants factor and Encounter Type (Reading Aloud, Reading Silently, and Listening) as a within participant factor. The ANOVA yielded a significant main effect of Encounter Type, *F*_(2, 418)_ = 4.76, MSE = 0.734, *p* = 0.009, η^2^_*p*_ = 0.02, and no significant main effect of Sample (*p* = 0.34) or interaction of Sample and Encounter Type (*p* = 0.61). As in the individual sample analyses, the only significant difference was between listening and reading aloud, *t*_(210)_ = 3.14, *p* = 0.002, 95% CI [−0.42 to −0.10], *r* = 0.21. Thus, it appears that listening to someone else read diminishes one's interest in the material, at least relative to reading aloud.

### Correlational analyses

Our study design also allowed us to examine a number of correlational findings that have previously been reported, as shown in Table 1. Consistent with previous research showing a negative relation between mind wandering and memory test performance (Schooler et al., 2004; Smallwood et al., 2008; Franklin et al., 2011; Risko et al., 2012), we found similar negative relations when Listening and Reading Silently, replicating these findings. Several researchers (e.g., Giambra and Grodsky, 1989; Krawietz et al., 2012; Unsworth and McMillan, 2013) have also previously found a negative relation between mind wandering and interest in the material and, as one would expect, we found significant negative correlations of Proportion of Mind Wandering and Interest Rating for each Encounter Type, in each sample. Interestingly, while these negative relations previously reported in the literature were replicated well in our samples, it is worth noting that a significant negative relation was not found between mind wandering and memory test performance when participants read aloud. Similarly, participants reading aloud showed nominally the weakest relation of mind wandering and interest in the material. At the same time, when participants listened to the excerpt the nominally largest relations with mind wandering were observed, and when they read silently more moderate relations were observed.

**Table 1:** **Pearson product-moment correlations of Proportion of Mind Wandering (Mind Wandering), Memory Test Proportion Correct (Memory) and Interest Rating (Interest), for each Encounter Type and sample**.

	**Sample 1**		**Sample 2**	
**Encounter type**	**Memory**	**Interest**	**Memory**	**Interest**
**READING ALOUD**				
Mind wandering	−0.08	−0.20^*^	0.01	−0.36^***^
Memory		0.14		0.23^*^
**READING SILENTLY**				
Mind wandering	−0.36^***^	−0.43^***^	−0.22^*^	−0.42^***^
Memory		0.40^***^		0.33^***^
**LISTENING**				
Mind wandering	−0.25^**^	−0.51^***^	−0.43^***^	−0.65^***^
Memory		0.33^***^		0.43^***^

*** p < 0.001,

*** p = 0.01,

* p < 0.05.

## Discussion

The present study broadly supports the notion that a more physically engaged reading experience means readers are likely to spend less time mind wandering. In two samples participants reported greater mind wandering when they were simply listening to another individual read, compared to when they engaged in more active forms of information consumption, namely reading silently and aloud. Interestingly, participants also reported less mind wandering when they were reading aloud than when they were reading silently, making reading aloud the most effective at preventing mind wandering. An additional consequence of these different types of engagement was decreased memory performance, such that more passively listening resulted in significantly worse memory for the material than either actively reading aloud or reading silently. In this regard it is interesting that, although reading aloud was able to reduce mind wandering relative to reading silently, there appeared to be no associated cost with respect to memory performance. Similarly, participants' interest in the material also showed no difference between reading silently and aloud, though simply listening, once again, resulted in less interest than when reading aloud.

During the review process we were made aware of a newly published study employing both aloud and silent reading conditions (Franklin et al., 2013). Interestingly, this study showed a greater proportion of self-reported mind wandering when reading aloud (0.32) than when reading silently (0.21)—an outcome that is numerically the opposite of the current findings. Such findings highlight that additional research on the effects of reading aloud is clearly necessary. There are, however, some notable differences between the two studies that are capable of accounting for these disparate findings. First, in the study by Franklin and colleagues, participants read their passage one sentence at a time, and had to advance sentences manually. This form of reading, though common for laboratory experiments, inevitably creates brief temporal gaps between each sentence and is quite different from normal reading outside of the laboratory. It may be the case that such a reading style is in fact different enough to reverse the effect of reading aloud vs. silently—particularly as it would at least momentarily disrupt the natural rhythm of speech when transitioning from one sentence to the next. Thus, the extended gap between sentences might afford additional opportunity for the participant's mind to wander. Alternatively, the reader may also be more likely to take notice of how odd it sounds to read sentences one at a time, and subsequently report these thoughts as mind wandering. A second likely explanation for the different findings between these two studies is that Franklin and colleagues used a between-subjects experimental design, while the current study used a within-subjects design. Research on the production effect has found that differences in memory performance for words read silently and words read aloud are generally only detected in within-subjects experimental designs (MacLeod et al., 2010), potentially owing to differences in encoding that only arise when the participant experiences both conditions. This suggests it may be critically important to use within-subjects experimental designs when comparing aloud and silent reading. In addition, between-subjects designs are more susceptible to both null outcomes and unusual outcomes if random assignment fails to equate the two groups, especially for studies using a small number of participants. Indeed, a between-subjects analysis of only the silent and aloud reading conditions in the current data set, using only the first reading session for each participant, reveals no significant difference for either sample. Notably, each sample is roughly equivalent in size to the sample collected by Franklin and colleagues (Ns = 71 and 70 for Samples 1 and 2, respectively). Clearly, then, more research is needed to address these alternative reasons for the differences between the outcomes of the two studies.

Despite finding different outcomes from Franklin et al. (2013) when reading aloud, we have nonetheless broadly replicated several previous findings of negative relations between mind wandering reports and memory test performance when reading silently and listening (Schooler et al., 2004; Smallwood et al., 2008; Franklin et al., 2011), though no correlation of memory test performance and mind wandering was observed when participants read aloud. Likewise, significant negative correlations between mind wandering reports and interest in the material were also observed for each type of encounter, and yet these relations were again the lowest when reading aloud and highest when simply listening to the material. In the case of the non-significant correlation of mind wandering and memory when reading aloud, it is possible, though perhaps not probable, that mind wandering was so infrequent when reading aloud, and memory for the material so good, that it simply may not have been possible to obtain enough systematic variation to observe a significant relation of mind wandering and memory. Nonetheless, as the effects of reading aloud on mind wandering have not previously been studied, these analyses clearly do replicate previous findings.

Our findings raise an interesting question, namely: why is it that under typical reading conditions the rate of mind wandering systematically decreases from listening, to silent reading, to reading aloud? We suggest that the key difference between these conditions is the extent to which physical activity is involved in the encounter with the material. Specifically, reading silently requires oculomotor activity that is not involved in listening, and reading aloud involves oculomotor activity *and* overt verbalization, the latter of which is not involved in silent reading. This difference in bodily involvement across conditions can influence mind wandering in at least two ways. One possibility is that an increase in bodily involvement might make the task more cognitively effortful and difficult. Since it is fairly well established that more difficult tasks lead to less mind wandering (Smallwood et al., 2007; Forster and Lavie, 2009; Thomson et al., 2013), the progressive increase in difficulty from listening, to silent reading, to reading aloud might lead to progressively less mind wandering. Another possibility is that the more the body is engaged in a task, the more cues are available to signal when the mind is going off task. For instance, the cessation of overt verbalization while reading aloud would be a very strong cue that attention has wandered away from the task; of course this cue is not available during either listening or silent reading. Both of these possibilities are consistent with the increasingly popular notion of embodied cognition (e.g., Clark, 1999; Wilson, 2002; Kingstone et al., 2008) in which mental activity is facilitated, reflected, and constrained by overt body behavior. Indeed, some of our own recent work converges on a similar point, having shown that blink rate increases when one is mind wandering, presumably to reduce external stimulation and facilitate directing thought internally (Smilek et al., 2010), and that mind wandering is associated with both self-reported (Carriere et al., 2013) and observed fidgeting behavior (e.g., Farley et al., 2013; Seli et al., in press).

Regardless of the mechanism involved, the present findings certainly show that different types of reading encounters can also entail important differences in mind wandering, memory performance, and interest in the material. These findings have important implications as new technology affords individuals more choices when selecting the methods by which they obtain information (e.g., podcasts, audiobooks, and eBooks), many of which tend to offer a less physically engaging experience. As technological advances continue to broaden our options for information consumption, it is important to recognize the strengths and weaknesses of each new method as it is developed. While listening to an audiobook or podcast may seem to be a convenient and appealing option, our findings suggest that it might be the least beneficial to learning, leading to both higher rates of mind wandering and less interest in the material.

### Conflict of interest statement

The authors declare that the research was conducted in the absence of any commercial or financial relationships that could be construed as a potential conflict of interest.

## Footnotes

^1^Here we distinguish between “listening” and “hearing,” as in Schnell (1995): “Hearing is merely a physiological process whereby you are aware of noise in the environment that does not require interpretation… Listening is an interpretive process whereby you make sense out of the sounds you are hearing” (p. 1).

^2^An analysis of data collected from only the first five probes for each participant and each reading encounter in Sample 1 revealed only nominal differences from analyses that included all recorded probes. Thus, the calculation of proportions was effective in accounting for missing probes, while allowing all available data to be used.

## Appendix

### Memory test questions

#### Chapter 11

1. The British scientist C. T. R. Wilson was studying quarks. (F)
2. An artificial cloud chamber cools and moistens air. (T)
3. Pions, mesons, intermediate vector bosons and tachyons are examples of particles discovered as a result of high-speed crashes in an atom smasher. (T)
4. Black holes and “strange quarks” are the typical result of particle collision. (F)
5. Enrico Fermi was largely responsible for naming the newly discovered particles from atom smashing. (F)
6. Scientists attempt to trap neutrinos in heavy water held in underground chambers. (T)
7. Neutrinos do not possess mass, which is why they can harmlessly pass through us and the Earth. (F)
8. The Superconducting Supercollider is the world's largest existing particle accelerator. (F)
9. Richard Feynman believed that we had discovered most of the particles in nature. (F)
10. The term “hadron” references protons, neutrons and other particles governed by strong nuclear force. (T)

#### Chapter 17

1. The atmosphere causes particles to slow as they entire, without it raindrops would pound us senseless. (T)
2. The atmosphere is very thick, if the Earth shrunk down to a desktop globe size the atmosphere would be about a foot deep. (F)
3. The four layers of the atmosphere include the: troposphere, biosphere, stratosphere and hydrosphere. (F)
4. The tropopause was discovered by a Frenchman in a balloon. (T)
5. The temperatures in the four layers of the atmosphere range from −130–2700° Fahrenheit. (T)
6. Only travelling very high into the atmosphere will affect bodily function and ability. (F)
7. Areas above 25,000 feet are known to climbers as DemiseZones. (F)
8. The ability to overcome bodily distress at extreme heights is mostly dependent on an individual's fitness level. (F)
9. Temperatures drop about 15 degrees Fahrenheit with every thousand feet you climb. (F)
10. Sunlight energizes atoms, allowing us to feel heat. (T)

#### Chapter 21

1. Fossils usually stay stable, so it is not difficult for them to maintain an identifiable shape. (F)
2. Approximately one bone in a million becomes fossilized. (F)
3. Estimates say about 80 species in ten thousand have made it into fossil record. (F)
4. About 95 per cent of all the fossils we possess are of animals that once lived under water. (T)
5. Richard Fortey was the first man to discover a human fossil. (F)
6. Finding a complete trilobite fossil is a common occurrence for a palaeontologist. (F)
7. Trilobites survived only 1 per cent as long as humans have. (F)
8. The size of trilobites ranges from the size of modern beetles, to as big as platters. (T)
9. Despite his active field research, Walcott only published one book during his career. (F)
10. Walcott was a founding director the advisory committee for Aeronautics, which eventually became the National Aeronautics and Space Agency, or NASA. (T)
